# Five Reasons Why I Am Skeptical That Indirect or Unconscious Lie Detection Is Superior to Direct Deception Detection

**DOI:** 10.3389/fpsyg.2019.01354

**Published:** 2019-08-16

**Authors:** Timothy R. Levine

**Affiliations:** Department of Communication Studies, University of Alabama at Birmingham, Birmingham, AL, United States

**Keywords:** lying, deception detection, truth-default theory, indirect detection, unconscious detection

## Abstract

The relative advantage of indirect and unconscious lie detection compared to direct detection is examined. Empirical evidence for the superiority of indirect and unconscious lie is unconvincing. Three empirical issues include comparisons of incommensurate outcomes, questionable results in control conditions, and evidence for improved performance of direct detection under some conditions. Two theoretical reasons for skepticism include consideration of the casual forces producing poor accuracy and the tendency for people to believe other people absent active cognitive processing. Generally speaking, in human lie detection, effortful and disciplined thought provides more accurate detection of lies than intuition or less than fully conscious cognitive processing.

Imagine a citizen watching a politician denying involvement in a scandal. Let us presume that the citizen’s interests are best served by knowing the truth. Should they carefully assess the politician’s verbal and nonverbal behaviors for deception cues? Might the citizen be better off relying on reputable journalistic fact-checking resources and applying critical thinking to the best available information? Or, might their intuition prove superior to active investigation or deliberation? Could it be that people’s subconscious minds are the best lie detectors?

Some researchers have made the twin claims that direct lie detection is poor and that indirect (e.g., DePaulo et al., 1997; Vrij et al., 2001) and/or unconscious lie detection (e.g., Reinhard et al., 2013; Ten Brinke et al., 2014) is superior to direct detection. This essay details five compelling reasons why we should be skeptical of these claims. First, some claims involve flawed apples-to-oranges comparisons involving different metrics of evaluation. Second, some evidence for unconscious lie detection involves a methodological issue I call “exploitation of aberrant controls.” Third, the ephemeral nature of cues makes cue- and demeanor-based lie detection necessarily error prone, unconscious or otherwise. Fourth, direct lie detection is only poor under certain limited conditions. Well-known meta-analytic claims are now dated. At best, any advantage for unconscious processes is situation specific. Finally, theory tells us that the human unconscious is a believer, not a skeptic. Both theory and data suggest that critical thinking and evidence, not intuition, are the best defenses against being duped. In the absence of conscious consideration otherwise, people are prone to believe other’s lies.

Before getting into the details, let me be explicit about my claims. I dispute that indirect or unconscious detection is always, necessarily, or typically superior to direct detection. I do not claim that direct detection is always effective. Direct detection can be chance-level or worse. I have designed experiments that reliably produce below-chance accuracy (e.g., Levine et al., 2011). I do claim that (1) direct detection is not always or necessarily poor and (2) indirect or unconscious detection is not inherently superior to direct detection. Can unconscious lie detection outperform direct detection under certain conditions that are not currently understood? Maybe. I do not know. Some convincing affirmative evidence favoring less conscious detection exists. Reinhard et al.’s (2013) series of experiments appear to provide compelling evidence that less conscious detection can outperform direct direction detection. Nevertheless, there are also good theoretical and empirical reasons for skepticism.

## A Shell Game of Metrics

At minimum, any assessment of relative advantage needs to apply common metrics or standards to the competitors. Direct detection is most often assessed on the metric of raw percent correct truth-lie classification. On this metric, the average accuracy produced in nearly 300 tests prior to 2006 is between 53 and 54% (Bond and DePaulo, 2006). The 54% finding seems, on face, to be poor. And, this is how direct detection accuracy is conventionally understood by social scientists. The humans-are-poor-lie-detectors claim is repeated in virtually every academic article on the topic over the past decade.

Some researchers have scaled honesty rather than using dichotomous, forced-choice, truth-lie items. In such cases, results are not scored as a percent correct like a true-false test. Instead, mean honesty ratings are compared for truths and lies. Researchers employing scaling have consistently found that honest messages are rated as more honest than lies, *p* < 0.05. They conclude that people can indeed detect deception when it is present, and some have gone farther to argue that poor accuracy is an artifact of direct dichotomous measurement (e.g., Burgoon et al., 1995). The evidence clearly shows that people can directly detect lies at *p* < 0.05 when honesty is directly scaled.

See the problem here? In the Bond and DePaulo (2006) meta-analysis, the 54% accuracy was greater than chance with a highly significant (*p* < 0.00001) *t* of nearly 40. The effect size was *d* = 0.41. So, direct dichotomous measurement of accuracy is significantly better than chance too. In the Bond and DePaulo meta-analysis, the effect size for dichotomous judgments is actually slightly larger than that for rating scales (*d* = 0.34). When evaluated on the same metrics of statistical significance and effect size, evidence for the superiority of scaling is revealed as illusory. Statistically, the null hypothesis of chance-rate accuracy is rejected with both types of direct (dichotomous and scaled) detection measures.

The flawed argument structure goes like this. Significant direct accuracy (54%) is re-cast as no better or only slightly better than chance and then contrasted with a statistically significant finding produced by an alternative method. Superiority is erroneously claimed. This is a straw man argument in which direct assessment is depicted as worse than it really is (no better than mere chance) and the illusion is created by comparing two different metrics: raw percentage which looks poor versus significance tests which are highly significant and replicate.

This problem also applies to many claims about indirect assessment. For example, DePaulo et al.’s (1997) confidence meta-analysis found that judges were significantly more confident (*d* = 0.30) in assessments of truths than lies. They concluded that “measures of indirect deception detection hold great promise” and “judges who appear to be totally unable to distinguish truths from lies based on their explicit judgments may show some evidence of accurate discrimination based on indirect measures” (p. 355) such as confidence ratings. But, a statistically significant effect size of *d* = 0.30 is not better than the findings for explicit judgments which approximate a statistically significant effect size of *d* = 0.40.

Bond et al. (2015) provided a meta-analytic test of accuracy from indirect assessment. Combined with the findings of Bond and DePaulo (2006), the best available evidence suggests that people distinguish between truths and lies at rates significantly better than chance with both direct and indirect measures, but the effect sizes are not systematically larger with indirect measures. Direct, dichotomous measures tend to yield directionally larger effect sizes than either scaled direct assessments or the majority of indirect measures, especially those measures that have been studied more often and have more stable mean effect sizes. Arguments for the superiority of indirect or scaled measures may be flawed when comparisons involve different metrics. Meta-analyses show such arguments to be empirically false when the assessment is based in comparable metrics.

## Exploitation of Aberrant Controls

Other evidence for unconscious lie detection rests on head-to-head experimental comparisons (e.g., Reinhard et al., 2013; Ten Brinke et al., 2014). In experiments with controls, differences between experimental groups are typically attributed to improvement in the treatment group. Differences, however, can also result from unusually low scores in the controls. Exploitation of aberrant controls occurs when a difference between a treatment and a control is, at least in part, a function of unusually poor performance in the control rather than improvement in the treatment (Levine and Bond, 2014; Levine et al., 2017). This appears to be the case in some studies of unconscious detection.

Ten Brinke et al. (2014) write: “Across two experiments, indirect measures of accuracy in deception detection were superior to traditional, direct measures. These results provide strong evidence for the idea[s] that although humans cannot consciously discriminate liars from truth tellers, they do have a sense, on some less-conscious level, of when someone is lying” (p. 1103). In Ten Brinke et al.’s (2014) first experiment, accuracy was (relative to chance) *d* = −0.23 in the control compared to *d* = +0.32 in the indirect condition. In experiment two, the effect size for direct detection was *d* = −0.01 compared to *d* = +0.27 for indirect detection. While indirect detection outperformed direct detection head-to-head in these two studies, the indirect effects underperformed the typical direct effect in meta-analysis. Compared to the literature as a whole, what is notable in the two experiments is the unusually poor performance in the control groups rather than unconscious detection being especially accurate. Logically, if the 54% accuracy corresponding to an effect size of *d* = +0.40 is considered poor accuracy, then effect sizes of +0.27 and + 0.32 must also be interpreted as poor.

It is my opinion that studies reporting underperforming controls are a potential cause for concern. Unusually poor accuracy can occur by chance (within-cell sampling and measurement errors) or by systematic factors such as using idiosyncratic senders with negative transparency (e.g., the use of poorly demeaned honest senders such as persons with social anxiety or on the autism spectrum). Whether produced by systematic or random factors, atypical findings, by definition, tend not to replicate. And, the indirect or unconscious detection literature has been characterized by replication failures (Street and Vadillo, 2016; Wu et al., 2019). Aberrant control findings are early warning signs of future failures to replicate.

## Cues are Ephemeral

Is there a true and valid signal for the human unconscious to use for accurate lie detection? I argue that the available evidence suggests that the answer is no (see Levine, 2018b). If people are poor lie detectors in some lie detection task, the cause may reside in the lack of a reliable and valid signal from the sender, a failure of a receiver to recognize and effectively utilize the signal, or both. The preponderance of evidence to date suggests that poor lie detection results more from a lack of valid signal on the sender end rather than deficient signal detection by receivers (Hartwig and Bond, 2011).

The reason that demeanor and cue-based lie detection is poor is simply that behavioral cues to deception are ephemeral (Levine, 2018b, 2019). Verbal and nonverbal cues vary from person to person, situation to situation, and message to message. There are large situational, individual, and intra-individual differences in senders that produce high error rates and push accuracy down toward chance (Levine et al., 2011; Levine, 2014, 2019). Cues can be useful with hindsight but not in real-time detection because receivers cannot know in advance which cues will hold utility for any one message by a given person in a specific situation. From this perspective, low accuracy is caused by a weak, inconsistent, and inherently error-producing signal. If this is true, direct and indirect, conscious and unconscious, detectors should all be poor for the same reason. There just is not a reliable signal to use consciously or otherwise in cue- and demeanor-based lie detection. Fortunately, real-time observation cues and demeanor are not the only way to detect lies.

## Direct is Not So Bad After All

Understanding the 54% average accuracy finding for direct detection requires much nuance. When used routinely and reflexively without extensive qualification, it can create a very misleading soundbite. The 54% average applies to variations on one particular experimental paradigm which methodologically constrains findings and produces very consistent results (Levine, 2018a). The research designs producing the 54% average do not model how people detect deception in everyday life (Park et al., 2002). Further, since 2006, there have been more than two dozen experiments reporting much improved direct lie detection performance using approaches other than cue- and demeanor-based lie detection (see Levine, 2015, 2019). The 54% average in direct detection holds for (1) real-time detection where (2) truths and lies are equally probable, (3) where evidence, content, motive, and persuasion-based direct strategies are all unavailable, and (4) where detection is based on observation of sender behavioral displays. At best then, unconscious lie detection might only have an advantage in the situations were direct accuracy is reliably poor. Further, the recent approaches that successfully improve lie detection all involve a mindful and deliberate approach to lie detection (see Levine, 2015, 2019 for reviews; see Reinhard et al., 2013 for an exception). There are, therefore, good reasons to believe that strategic, mindful approaches involving investigation and/or critical thinking are the fruitful path to better lie detection.

## The Human Unconscious is a Believer, Not a Skeptic

There are additional theoretical reasons to believe that active approaches to lie detection have much potential for improved lie detection while less-than-fully mindful humans are more easily duped. Truth-Default Theory (Levine, 2014, 2019) holds that humans are social beings whose success as individuals, collectives, and as a species depends on efficient cooperation, coordination, and communication among fellow humans, especially within in-groups. The truth-default (passive acceptance of incoming communication content) makes efficient cooperation, coordination, and communication possible. Absent something to actively trigger suspicion, doubt, or disbelief, people passively believe others. Gilbert’s (1991) Spinozian Belief Model makes similar claims. If Gilbert and Levine are right, disbelief and attribution of deception require deliberate cognitive processing.

## Future Directions

I am also skeptical that more research will provide a resolution anytime soon. Social scientists sometimes adopt a version of “naïve empiricism” in which mixed findings are presumably settled by the results of the next study. Often, however, more data just means more results that do not replicate. Consider the most recent studies of unconscious detection. Wu et al. (2019) failed to replicate Reinhard et al. (2013) in two experiments. Values of *F* were less than 1.00 and Bayesian analyses were more consistent with the null. Moi and Shanks (2015) report similar results to Wu et al. Which set of findings should we believe? Here we have five supportive findings and four disconfirming results.

My skepticism about the short-term utility of “more research” stems from two interrelated observations. First, the replication crisis in social science is, in my opinion, very real. Most findings even in the best journals do not replicate (Open Science Collaboration, 2015). Second, in my experience, most meta-analyses find unresolved heterogeneity. What this means is that often studies find different things and we simply do not know why or which (if any) to believe.

For the theoretical reasons articulated above, my own opinion about the ultimate outcome is that the unconscious detection hypothesis will eventually be abandoned. But, the only satisfying resolution will be to find a moderator that fully resolves the current heterogeneity in the literature. To uncover such a moderator, we likely need some new theory, some new insight, or maybe a little luck.

## Conclusion

I am skeptical that indirect or unconscious lie detection is generally, usually, or typically superior to direct deception detection. Arguments for the superiority of indirect lie detection often involve comparing statistical probabilities to raw percentages. When evaluated on the same statistical metric, meta-analysis shows that effect sizes associated with direct detection are directionally larger than those of indirect assessment. In experimental studies involving head-to-head comparisons, performance in the controls is sometimes inexplicably poor.

In situations where direct deception detection accuracy is poor, the reason for the poor performance stems from an unreliable signal rather than poor reception by the conscious mind. Because the cause of poor accuracy is on the stimulus side rather the reception end, indirect measurement is unlikely to solve the problem. The keys to improved lie detection involve seeking a better signal, not a different way of processing of an error-ridden signal.

I believe that accurate lie detection is possible under certain conditions, but that it has little to do correctly interpreting the verbal and nonverbal behavioral cues of communicators. Outside of the lab, most lies are detected after-the-fact based on evidence apart from sender behavior (Park et al., 2002). Fact-checking and critical thinking are better lie detection tools than the subconscious mind.

Humans have a strong truth-default when processing incoming communication. The tendency to passively believe others puts people at risk for deception. Abandoning the truth-default and correctly recognizing deception for what it is requires active and deliberate cognitive processing. The subconscious mind is not the best lie detector. Fact-checking and applying critical thinking to the available information are superior to intuition and less conscious processing.

## Author Contributions

The author confirms being the sole contributor of this work and has approved it for publication.

### Conflict of Interest Statement

The author declares that the research was conducted in the absence of any commercial or financial relationships that could be construed as a potential conflict of interest.
